# Global Prevalence of Burnout in Gastroenterology and Endoscopy: A Systematic Review and Meta‐Analysis

**DOI:** 10.1002/ueg2.70045

**Published:** 2025-06-17

**Authors:** Mohamed G. Shiha, Francesca Manza, John Ong, Iago Rodríguez‐Lago, Martina Müller, Reena Sidhu

**Affiliations:** ^1^ Division of Clinical Medicine School of Medicine and Population Health University of Sheffield Sheffield UK; ^2^ Department of Gastroenterology University Hospitals of Leicester Leicester UK; ^3^ Academic Unit of Gastroenterology Sheffield Teaching Hospitals Sheffield UK; ^4^ Department of Translational Medicine St. Anna Hospital University of Ferrara Ferrara Italy; ^5^ School of Clinical Medicine University of Cambridge Cambridge UK; ^6^ Department of Gastroenterology & Hepatology Bedfordshire Hospitals NHS Trust Bedford UK; ^7^ Department of Gastroenterology Hospital Universitario de Galdakao Biobizkaia Health Research Institute Deusto University Galdakao Spain; ^8^ Department of Internal Medicine I, Gastroenterology, Hepatology, Endocrinology, Rheumatology, Immunology, and Infectious Diseases University Hospital Regensburg Regensburg Germany

**Keywords:** burnout, depersonalisation, emotional exhaustion, endoscopy, gastroenterology, gender disparities, hepatology, personal accomplishment, physician well‐being

## Abstract

**Background:**

Burnout is an increasingly recognised phenomenon that negatively affects physicians' well‐being, patient safety and the sustainability of healthcare systems. In this systematic review and meta‐analysis, we aimed to estimate the global prevalence of burnout in gastroenterology and endoscopy.

**Methods:**

We searched Medline, Embase, Scopus and PsycINFO up to November 2024 for studies reporting the prevalence of burnout in gastroenterology and endoscopy. The primary outcome was the prevalence of burnout among gastroenterologists and endoscopists. Secondary outcomes included the prevalence of emotional exhaustion, depersonalisation, low sense of personal accomplishment and gender‐related differences in burnout. We used random‐effects models to calculate the pooled prevalence and odds ratios (OR) with their 95% confidence intervals (CI).

**Results:**

A total of 22 studies comprising 8124 participants were included. The pooled prevalence of burnout was 45% (95% CI, 37%–54%), with significant variability observed across different geographical regions, assessment tools and definitions of burnout. The pooled prevalence of emotional exhaustion was 31% (95% CI, 22%–40%), depersonalisation was 23% (95% CI, 16%–31%), and low sense of personal accomplishment was 25% (95% CI, 10%–40%). Female gastroenterologists were more likely to experience burnout than males (OR 1.53; 95% CI, 1.16–2.01; *p* < 0.001).

**Conclusions:**

Almost half of gastroenterologists and endoscopists experience burnout, with females being disproportionately affected. These findings highlight the need for urgent action to address burnout and its contributing factors, including gender disparities.

## Graphical abstract

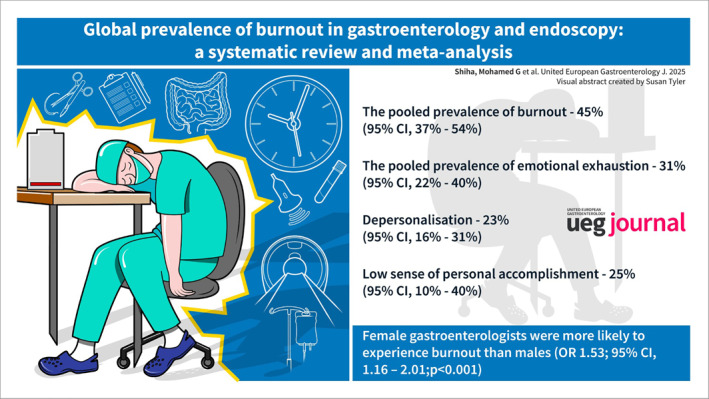

1

**Summary**

- Summarise the established knowledge on this subject
  ◦ Burnout is common among healthcare professionals, with significant implications for mental health, job satisfaction, and patient care.
  ◦ Gastroenterologists and endoscopists are at risk of burnout due to high workloads, long hours, and the demanding nature of their roles.
  ◦ Previous studies have reported varying prevalence rates of burnout among gastroenterologists and endoscopists. However, comprehensive global estimates remain unavailable.
- What are the significant and/or new findings of this study?
  ◦ Burnout approximately affects half of gastroenterologists and endoscopists, with wide variations observed across different geographical regions, assessment tools and definitions of burnout.
  ◦ Female gastroenterologists and endoscopists have 53% increased odds of burnout compared with their male counterparts.

## 2 Introduction

Physician burnout is an occupational phenomenon resulting from chronic stress within the workplace. It is characterised by emotional exhaustion, depersonalisation and a low sense of personal accomplishment. Emotional exhaustion refers to the constant feeling of energy depletion that leaves physicians drained and unable to meet the demands of their clinical duties. Depersonalisation involves a marked sense of detachment from work and a lack of empathy towards colleagues and patients, who are perceived as obstacles. The third dimension of burnout is the low sense of personal accomplishment, which reflects the feeling of ineffectiveness and lack of professional achievement, further exacerbating job dissatisfaction [1].

Burnout among physicians is increasingly recognised worldwide, with prevalence rates ranging from 0% to 80.5% depending on the population and definitions used [2]. The consequences of burnout do not only impact physicians' mental health and well‐being but can profoundly affect patient care and the overall function of healthcare systems. Physicians experiencing burnout are more prone to making significant medical errors and are at a higher risk of being named in medical malpractice claims [3]. Moreover, physician burnout has been directly linked to adverse patient outcomes, including reduced satisfaction, lower quality of care and longer post‐discharge recovery times [4].

Gastroenterologists and endoscopists are particularly prone to experiencing burnout due to the high‐pressure nature of their work, which involves complex procedures, long hours, and managing patients with chronic and life‐threatening conditions [5]. Indeed, previous studies have shown that burnout is common among gastroenterologists, with prevalence rates ranging from 18.3% to 64.4% [6]. In this systematic review and meta‐analysis, we aimed to provide comprehensive data on the global prevalence of burnout in gastroenterology and endoscopy and to identify key risk factors for burnout.

## 3 Methods

We conducted this systematic review and meta‐analysis in accordance with the Preferred Reporting Items for Systematic Reviews and Meta‐analyses (PRISMA) guidelines [7] (supplementary material). The study protocol was prospectively registered on the International Prospective Register of Systematic Reviews (PROSPERO, CRD42024598138; 5th November 2024).

### 3.1 Data Sources and Search Strategy

We systematically searched Medline, Embase, Scopus, and PsycINFO from their inception to the 27th of November 2024 for studies reporting the prevalence and risk factors of burnout in gastroenterology and endoscopy. The search strategy was developed in collaboration with an expert medical librarian and is provided in supplementary material. There were no language restrictions; studies not published in English were translated using Google Translate. A recursive search of the bibliographies of all eligible articles and reviews was performed to identify any relevant studies not captured by the database search.

### 3.2 Study Selection and Eligibility Criteria

The search results were exported to EndNote 20 (Clarivate Analytics, London, United Kingdom), and duplicate records were removed. Two reviewers (MGS & FM) independently screened the titles and abstracts for potentially relevant studies. We included observational studies that assessed the prevalence and risk factors of burnout among gastroenterologists, hepatologists and endoscopists, including fellows and trainees. Studies that provided quantitative data on the prevalence of burnout or its three dimensions (emotional exhaustion, depersonalisation and low sense of personal accomplishment) were eligible for inclusion. Only full‐text publications were considered. We excluded conference abstracts, case reports, reviews, editorials, practice guidelines, and studies that lacked specific data on burnout outcomes.

The full‐text articles of all eligible studies were retrieved and evaluated in more detail against the inclusion criteria by the two reviewers, with any discrepancies resolved by consensus.

### 3.3 Data Extraction

Two reviewers (MGS & FM) independently extracted data from the included studies onto a standardised Excel spreadsheet (Microsoft Corp, Redmond, WA). Where available, the following data were extracted from each study: study authors, year of publication, study period, country, population, sample size, participant demographics, burnout assessment methods, and relevant study outcomes. Disagreements between reviewers were resolved by consensus. We contacted the corresponding authors where additional information was needed.

### 3.4 Quality Assessment

The quality of the included studies was assessed using a modified Newcastle‐Ottawa Scale (NOS) for cross‐sectional surveys [8]. Two reviewers (MGS & FM) independently evaluated the studies using the modified NOS tool based on the following domains: selection of participants, adjusting for confounding factors, and the assessment of outcomes. The quality of each study was classified as unsatisfactory (0–4 points), satisfactory (5–6 points) or good (≥ 7 points). The modified NOS tool is provided in the supplementary material.

### 3.5 Data Synthesis and Statistical Analyses

We calculated the pooled prevalence of burnout, emotional exhaustion, depersonalisation and low sense of personal accomplishment with their 95% confidence intervals (CI) using a random‐effects model meta‐analysis to account for between‐study heterogeneity. Additionally, we performed several subgroup analyses based on burnout assessment tools, response rates, career stage, study region and survey period. We also performed sensitivity analyses by excluding studies using non‐validated tools to assess burnout. Finally, we conducted a leave‐one‐out analysis by excluding each study one at a time and recalculating the pooled prevalence of burnout to determine the influence of each study on the overall effect size estimates. The odds ratio (OR) for burnout in female gastroenterologists and endoscopists compared with their male counterparts was calculated to evaluate gender differences. Heterogeneity was assessed using *I* ^2^ statistics, with *I* ^2^ values of 25%, 50%, and 75% considered low, moderate, and high heterogeneity, respectively. We assessed publication bias using funnel plots and Egger's test. A *p*‐value of < 0.05 was considered statistically significant. All statistical analyses were performed using Stata version 18 (StataCorp, College Station, Texas, USA).

## 4 Results

### 4.1 Study Selection and Characteristics

**FIGURE 1:**
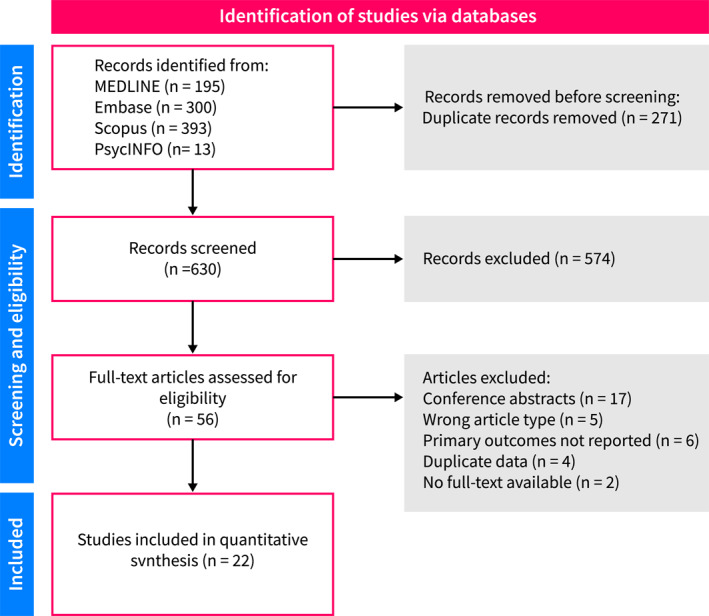
PRISMA flow diagram of study selection.

Our database search yielded 901 results. After excluding duplicates and non‐relevant studies, 56 studies were eligible for full‐text screening. Of these, 22 studies comprising 8124 participants met the inclusion criteria and were included in the final analyses [9, 10, 11, 12, 13, 14, 15, 16, 17, 18, 19, 20, 21, 22, 23, 24, 25, 26, 27, 28, 29, 30] (Figure 1).

The characteristics of the included studies are summarised in Table 1. All the included studies were cross‐sectional surveys of gastroenterologists, hepatologists and endoscopists at different career stages. The studies were conducted between 1996 and 2024 across multiple regions, including Europe, Asia, North America, South America, Africa, and Oceania. The response rates varied widely, ranging between 5% and 93.2%. Most studies used validated tools to assess burnout, such as the Maslach Burnout Inventory (MBI) and the Copenhagen Burnout Inventory (CBI), while a smaller number relied on the single‐item burnout score or non‐validated measures. Two studies [16, 19] were post hoc analyses of the Pawlak et al. study [15]. One study reported the prevalence of burnout using both MBI and single‐item scores; we used the MBI score in the primary analysis for consistency [11]. Anderson et al. study included two surveys which were analysed separately [26].

**TABLE 1:** Study characteristics.

Authors, year	Country	Survey period	Study participants	Sample size	Male (%)	Mean/median* age (years)	Response rate (%)	Burnout assessment tool	Modified Newcastle‐Ottawa score
Ramirez et al., 1996	UK	NR	Gastroenterologists	241	NR	NR	80.6	MBI	7
Keswani et al., 2011	USA	NR	Gastroenterologists	410	85.6	45	6.9	MBI	7
Aguilar‐Nájera et al., 2020	Mexico	April–November 2018	Members of MGA & MAGE	411	47.9	NR	22.9	MBI	8
Jang et al., 2020	South Korea	April–October 2019	Gastroenterologists	222	55.9	NR	NR	MBI	7
Ong et al., 2020	UK	January–February 2020	Gastroenterology trainees	40	70	33.1	44	MBI	6
Adarkwah et al., 2020	Germany	January–April 2019	Gastroenterologists	683	74.4	48.3	21.9	MBI	8
Pawlak et al., 2020^a^	International	April–May 2020	Endoscopy trainees	666	56.9	NR	NR	Single‐item burnout score	7
Siau et al., 2020^a^	UK	April–May 2020	Gastroenterology trainees	120	NR	NR	NR	Single‐item burnout score	5
Demirtaş et al., 2021	Turkey	October 2020	Gastroenterology trainees	96	81.3	34*	93.2	Non‐validated questionnaire	6
Correia et al., 2021	Portugal	February–April 2019	Gastroenterologists	52	46.2	44.9	9.0	CBI	6
Khan et al., 2021^a^	Canada	April–May 2019	Gastroenterology trainees	34	58.8	31*	39.1	Single‐item burnout score	5
Kriss et al., 2021	USA	May–June 2019	Transplant hepatologists	124	NR	38*	48.4	Single‐item burnout score	8
Ong et al., 2021	Southeast Asia	September–December 2020	Gastroenterologists	683	57.8	NR	38.8	MBI	8
Sanguinetti et al., 2021	Argentina	September–November 2020	Endoscopists	208	66.8	48.5	19.8	Non‐validated questionnaire	4
Pourmand et al., 2022	USA	October–December 2019	Transplant hepatologists	185	61	NR	12.7	MBI	7
Russo et al., 2022	USA	February–March 2021	Gastroenterologists and hepatologists	230	NR	59.3	9.6	MBI	7
Yuan et al., 2022	China	2020	Gastroenterologists	1655	31.6	39.2	NR	MBI	7
Anderson et al., 2023	USA	Survey 1 (2014–2015)	Gastroenterologists	756	78.7	52.3	6.8	MBI	8
		Survey 2 (December 2019–March 2020)	Gastroenterology fellows	323	66.9	32.5	26	MBI	
Chien et al., 2023	USA	February–March 2020	Paediatric gastroenterologists	406	NR	NR	22.7	2 single‐item measures	7
David et al., 2023	Europe	April–October 2021	ECCO IBD specialists	102	56.9	48	5	MBI	6
Dranga et al., 2023	Romania	NR	Gastroenterologists	40	22.5	30*	44.4	MBI	6
Mishra et al., 2024	India & Pakistan	November 2021–March 2022	Gastroenterologists	591	86.3	41.1	NR	Non‐validated questionnaire	6

*Note:* Asterisk symbol in the table is added over the numbers that are medians, the others are means.

Abbreviations: CBI, Copenhagen Burnout Inventory; ECCO, European Crohn's and Colitis Organisation; IBD, inflammatory bowel disease; MAGE, the Mexican Association for Gastrointestinal Endoscopy; MBI, Maslach Burnout Inventory; MGA, the Mexican Gastroenterological Association; NR, not reported; UK, United Kingdom; USA, United States of America.

^a^ Studies with overlap in participants.

### 4.2 Prevalence of Burnout

**FIGURE 2:**
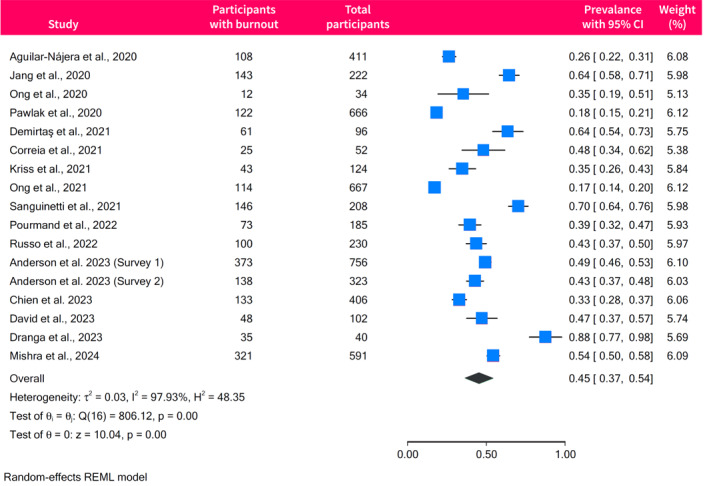
Forrest plot of the pooled prevalence of burnout among gastroenterologists and endoscopists.

The pooled prevalence of burnout among gastroenterologists and endoscopists was 45% (95% CI, 37%–54%), with high between‐study heterogeneity (*I* ^2^ = 98%) (Figure 2). Egger's test did not show evidence of small‐study effects (*p* = 0.17), and the funnel plot appeared symmetrical (Figure S1). The prevalence of burnout significantly varied according to the tool used for assessment (*p* < 0.001) (Figure S2). Studies using the 22‐item MBI scores had a pooled burnout prevalence of 45% (95% CI, 33%–57%; *I* ^2^ = 98%), which was similar to the prevalence reported in Correia et al. (48%; 95% CI, 34%–62%) who used the Copenhagen Burnout Inventory (CBI) [27]. Studies using the single‐item burnout score and the 2 single‐item measures had a lower prevalence of 26% (95% CI, 10%–42%; *I* ^2^ = 98%), and 33% (95% CI, 28%–37%), respectively. In contrast, studies using non‐validated tools to assess burnout reported the highest prevalence of burnout with pooled estimates of 62% (95% CI 53%–72%, *I* ^2^ = 86%) [17, 22, 30]. Excluding those studies did not significantly alter the overall estimates, with a pooled prevalence of 42% (95% CI, 32%–51%; *I* ^2^ = 98%) (Figure S3).

The prevalence rates varied significantly across different regions and countries (*p* < 0.001), as outlined in Table 2. However, there were no significant differences in the prevalence of burnout according to response rates (*p* = 0.30) (Figure S4), career stage (*p* = 0.41) (Figure S5), or the timing of studies in relation to the COVID‐19 pandemic (*p* = 0.55) (Figure S6).

**TABLE 2:** Prevalence of burnout among gastroenterologists and endoscopists by region and country.

	Studies (*n*)	Participants (*n*)	Prevalence (95% CI)	*I* ^2^
Europe	7	647	44% (24%–64%)	96.4%
UK	2	154	22% (0%–45%)	87.4%
Turkey	1	96	63.5% (54%–73.2%)	—
Romania	1	40	87.5% (77.3%–97.7%)	—
Portugal	1	52	48.1% (34.5%–61.7%)	—
Asia	4	1579	32.4% (19%–46%)	97.7%
India	1	476	51.9% (47.4%–56.4%)	—
Brunei	1	6	33% (0%–71.1%)	—
Indonesia	1	192	5.2% (2.1%–8.4%)	—
Malaysia	1	74	35.1% (24.3%–46%)	—
Pakistan	1	124	59.7% (51%–68.3%)	—
Philippines	1	134	11.9% (6.5%–17.4%)	—
Singapore	1	66	30.3% (19.2%–41.4%)	—
South Korea	1	222	64.4% (58.1%–70.7%)	—
Thailand	1	195	20.5% (14.8%–26.2%)	—
North America	8	2640	36.2% (29.4%–43%)	92.5%
USA	5	2024	40.7% (35.5%–48.7%)	81.4%
Mexico	1	411	26.3% (22%–30.5%)	—
Canada	1	34	29.4% (14.1%–44.7%)	—
South America	2	307	44.2% (0%–95%)	99%
Argentina	1	208	70.2% (64%–76.4%)	—
Africa	1	13	30.8% (5.7%–56%)	—
Oceania	1	40	2.5% (0%–7.3%)	—

Abbreviations: UK, United Kingdom; USA, United States of America.

Leave‐one‐out analysis confirmed that no individual study significantly influenced the overall estimates, with consistent prevalence rates ranging between 43% (95% CI, 35%–51%) to 47% (95% CI, 39%–56%) after sequentially excluding one study at a time (Figure S7).

### 4.3 Prevalence of Burnout Dimensions

**FIGURE 3:**
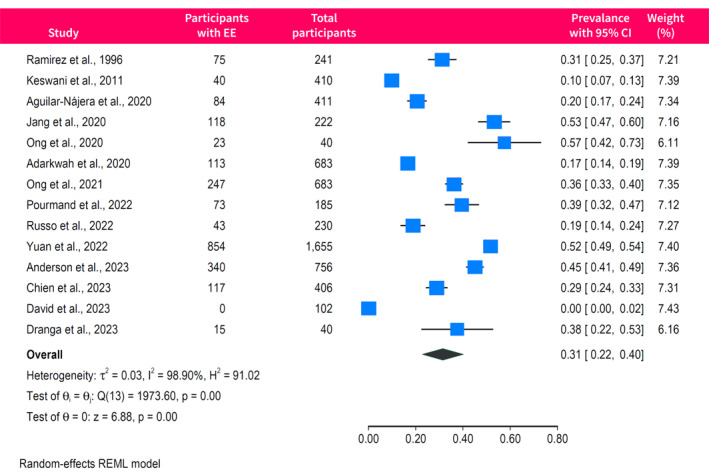
Forest plot of the pooled prevalence of emotional exhaustion among gastroenterologists and endoscopists. EE, emotional exhaustion.

**FIGURE 4:**
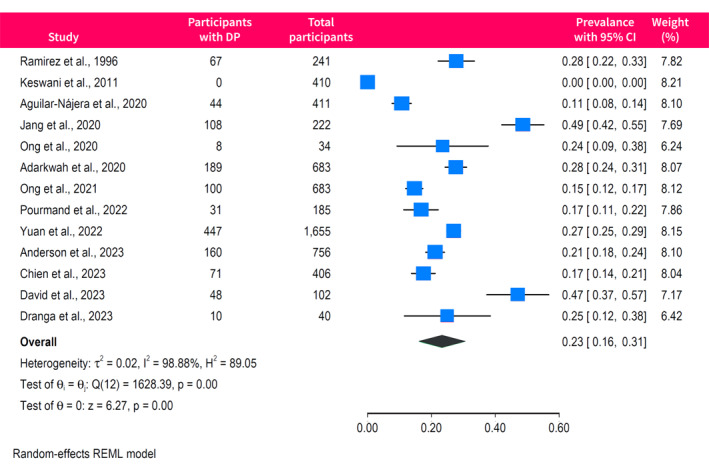
Forest plot of the pooled prevalence of depersonalisation among gastroenterologists and endoscopists. DP, depersonalisation.

**FIGURE 5:**
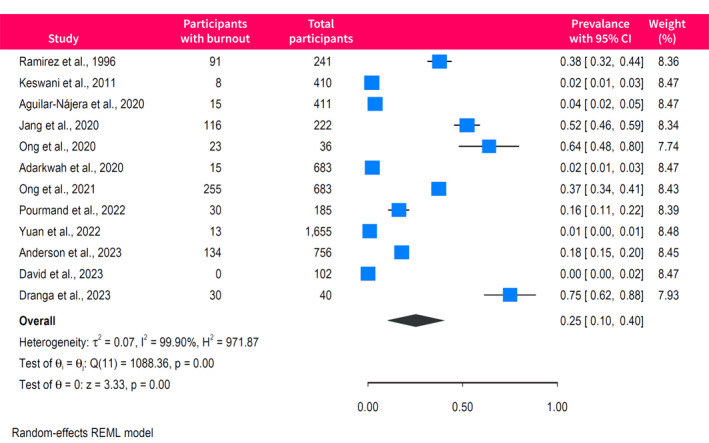
Forest plot of the pooled prevalence of low sense of personal accomplishment among gastroenterologists and endoscopists. LPA, low sense of personal accomplishment.

The rates of emotional exhaustion were reported in 14 studies with a pooled prevalence of 31% (95% CI, 22%–40%) and high between‐study heterogeneity (*I* ^2^ = 99%) (Figure 3). There was evidence of funnel plot asymmetry (Egger *p* = 0.04), indicating possible publication bias or small‐study effects (Figure S8). Depersonalisation was assessed in 13 studies, yielding a pooled prevalence of 23% (95% CI, 16%–31%; *I* ^2^ = 99%) (Figure 4), with no evidence of publication bias (Egger *p* = 0.06). Finally, 12 studies reported the rates of low sense of personal accomplishment, with a pooled prevalence of 25% (95% CI, 10%–40%; *I* ^2^ = 100%) (Figure 5). The funnel plot of studies assessing the low sense of personal accomplishment was asymmetrical (Egger *p* < 0.0001), indicating possible publication bias or small‐study effects (Figure S9).

### 4.4 Gender and Burnout

Gender differences in the prevalence of burnout were reported in 13 studies. Female gastroenterologists and endoscopists were more likely to experience burnout compared with their male counterparts (OR 1.53; 95% CI, 1.16–2.01; *p* < 0.001), with moderate between‐study heterogeneity (*I* ^2^ = 65.4%) (Figure S10). There was no evidence of funnel plot asymmetry (Egger *p* = 0.07) (Figure S11).

### 4.5 Quality Assessment

Overall, the quality of the included studies was good (*n* = 13) or satisfactory (*n* = 8). The modified NOS scores ranged between 4 and 8 with a median score of 7. Most studies included a representative sample and adjusted for potential confounders by subgroup or multivariate analyses. However, a common limitation was the low response rates (*n* = 6) and the use of non‐validated tools to screen for burnout (*n* = 3).

## 5 Discussion

In this systematic review and meta‐analysis of 22 studies including over 8000 participants, we found high prevalence rates of burnout among gastroenterologists, hepatologists and endoscopists. The pooled prevalence of burnout was 45% (95% CI, 37%–54%), with significant variability observed across different populations, geographical regions, assessment tools and definitions of burnout. The pooled prevalence of emotional exhaustion was 31% (95% CI, 22%–40%), depersonalisation was 23% (95% CI, 16%–31%), and low sense of personal accomplishment was 25% (95% CI, 10%–40%). Significant gender disparities were observed as female gastroenterologists and endoscopists were more likely to experience burnout compared with their male counterparts.

Our findings are consistent with reports from other high‐demand and interventional specialities. A previous meta‐analysis, including 8617 surgeons and surgical trainees, reported an overall burnout prevalence of 47% [31]. Similarly, a prospective cohort study of 4732 US resident physicians showed that training in general surgery, urology, neurology, emergency medicine and ophthalmology was associated with a higher risk of burnout compared with training in internal medicine [32]. A more recent survey of cardiology trainees in the United Kingdom reported a burnout prevalence of 76%, with half of the trainees stating that their training had a negative impact on their well‐being [33]. Gastroenterology and endoscopy share similar intense demands with long working hours, high procedural workloads, complex chronic disease management and extensive administrative tasks [26]. Therefore, the high prevalence of burnout among gastroenterologists is alarming but hardly surprising. Interestingly, despite heightened anxiety levels during the COVID‐19 pandemic, particularly among trainees, the prevalence of burnout did not significantly increase [15, 16, 17, 19]. This suggests that burnout stems from deeper, systemic causes such as sustained workload pressures, lack of organisational support, and chronic emotional exhaustion rather than acute stressors such as those experienced during the pandemic. Moreover, our findings revealed no significant difference in the prevalence of burnout between fully qualified gastroenterologists and trainees or fellows. This is likely due to the high workload faced by both groups, with trainees balancing clinical duties with endoscopy training and specialists managing complex cases and increasingly demanding responsibilities.

In our analyses, we observed differences in burnout prevalence depending on whether studies used the MBI tool, single‐question assessment, or other screening tools. The MBI, a comprehensive and widely validated tool, evaluates burnout across the three dimensions of emotional exhaustion, depersonalisation, and low sense of personal accomplishment, while single‐question assessments provide a more subjective and simplified measure. This methodological difference likely contributes to variability in reported burnout rates. Nonetheless, there is no universal agreement on the optimal tool for burnout assessment, which continues to pose a challenge in accurately measuring and comparing burnout rates across studies.

Female gastroenterologists and endoscopists are disproportionately affected by burnout. This aligns with previous studies that found female physicians across different disciplines to have up to 60% higher odds of burnout than males [3]. Gender disparity highlights the additional challenges faced by female gastroenterologists, such as balancing career progression with caregiving responsibilities, underrepresentation in leadership roles, and increased exposure to workplace bias and discrimination [34, 35]. Beyond gender, studies have identified other key risk factors for burnout, such as younger age, limited job autonomy, frequent on‐call shifts, and longer working hours [6, 18, 26, 27]. These challenges are further compounded by the increased use of electronic health records without adequate administrative staff support, which adds to the non‐clinical workload and detracts from patient care [27].

Interventions to tackle physician burnout have been extensively studied. Yet, many physicians may remain unaware of the available resources for seeking help. A meta‐analysis of 15 randomised controlled trials and 37 cohort studies involving 3630 physicians found that interventions such as mindfulness, stress management, small group discussion and duty hour limitations decreased overall burnout from 54% to 44% (*p* < 0.0001) [36]. We have provided a comprehensive summary of potential interventions for burnout in Table S1. Nonetheless, there is a lack of gastroenterology‐specific data on the effectiveness of these interventions. Given the high prevalence of burnout in gastroenterology, future studies should focus on developing and evaluating organisational‐ and individual‐focused interventions tailored to address the unique challenges of gastroenterology and endoscopy. Potential strategies include providing additional administrative staff support, allocating protected personal development and research time, introducing flexible working hours to improve work–life balance and reducing endoscopy lists to allow more time for procedures and patient care.

This study has several strengths. First, we adhered to the state‐of‐the‐art methodology for systematic reviews and meta‐analysis, including a priori registered protocol with clearly defined inclusion and exclusion criteria. Second, we performed a comprehensive literature search across several databases without time or language restrictions and translated foreign language articles to ensure all eligible articles were included. Third, study selection, data extraction, and quality assessment were independently performed by two reviewers. Fourth, we conducted extensive subgroup and sensitivity analyses to explore potential sources of heterogeneity and to assess the robustness of our results. Finally, the large sample size and wide geographical representation of the included studies increase the generalisability of our findings.

Our study also had several significant limitations. The inherent lack of a universally accepted assessment tool or definition for burnout led to substantial between‐study heterogeneity, which was not fully explained by our subgroup analyses. However, studies using the widely validated MBI tool had a pooled prevalence similar to the overall estimates, suggesting that our findings provide a reliable reflection of burnout prevalence despite the methodological variability in the included studies. The low response rates in some studies may have introduced selection bias as individuals experiencing burnout might have been more likely to participate. However, we found no significant differences in the pooled prevalence of burnout between studies with high and low response rates. There was a limited number of studies and participants from specific regions and countries, which may have resulted in underrepresenting certain healthcare systems or overestimating burnout prevalence in some regions. Lastly, as we did not have access to individual participant data, we were not able to provide granular data on contributing factors to burnout such as work hours, job autonomy, institutional support, or maternity leave duration.

In conclusion, this systematic review and meta‐analysis demonstrates that almost half of gastroenterologists and endoscopists worldwide experience burnout, with significant variations according to the definition of burnout, burnout assessment tools and geographical regions. Female gastroenterologists are at higher risk of burnout, highlighting the need for gender‐specific interventions to address unique challenges such as work‐life balance, workplace bias, and underrepresentation in leadership roles. Our findings emphasise the urgent need to address the alarming prevalence of burnout among gastroenterologists and endoscopists. Safeguarding gastroenterologists' well‐being is critical to maintaining a high standard of patient care and the overall sustainability of the speciality.

## Author Contributions

Conception: R.S., M.G.S. Literature search. Data extraction: M.G.S., F.M. Statistical analysis. Data visualisation: M.G.S. Quality assessment: M.G.S, F.M. Initial drafting of the manuscript: M.G.S., F.M. Data interpretation, critical revision of the manuscript. Final approval of the submitted version: all authors.

## Conflicts of Interest

M.G.S. is a Trainee Editor at UEG Journal. All the other authors declare no competing interests.

## Supporting information

Supporting Information S1

## Data Availability

Data are available upon reasonable request.
