# Supplementary material for: Global Prevalence of Burnout in Gastroenterology and Endoscopy: A Systematic Review and Meta‐Analysis
Source: United European Gastroenterol J. 2025 Jun 17;13(7):1318–27. doi: 10.1002/ueg2.70045 (PMC12463698; doi:10.1002/ueg2.70045)
Supplement: Supplementary file 1 — Supporting Information S1 [file UEG2-13-1318-s001.docx]

**Supplementary Materials**

Contents

[PRISMA Checklist 3](#_Toc191195495)

[Search Strategy 6](#_Toc191195496)

[MEDLINE 6](#_Toc191195497)

[Embase 6](#_Toc191195498)

[Scopus 7](#_Toc191195499)

[PsycINFO 7](#_Toc191195500)

[Modified Newcastle-Ottawa scale 9](#_Toc191195501)

[Supplementary Figures 10](#_Toc191195502)

[Supplementary Figure 1 – Funnel plot of studies reporting the prevalence of burnout among gastroenterologists and endoscopists 10](#_Toc191195503)

[Supplementary Figure 2 – Forest plot of studies reporting the prevalence of burnout sub-grouped according to the tool used for assessment 11](#_Toc191195504)

[Supplementary Figure 3 – Forest plot of studies reporting the prevalence of burnout excluding those using non-validated questionnaires 12](#_Toc191195505)

[Supplementary Figure 4 – Forest plot of studies reporting the prevalence of burnout sub-grouped according to response rates 13](#_Toc191195506)

[Supplementary Figure 5 – Forest plot of studies reporting the prevalence of burnout sub-grouped according to participants’ career stage 14](#_Toc191195507)

[Supplementary Figure 6 – Forest plot of studies reporting the prevalence of burnout sub-grouped according to the time of the survey 15](#_Toc191195508)

[Supplementary Figure 7 – Leave one out sensitivity analysis showing the pooled prevalence of burnout after excluding one study at a time 16](#_Toc191195509)

[Supplementary Figure 8 – Funnel plot of studies reporting the prevalence of emotional exhaustion among gastroenterologists and endoscopists 17](#_Toc191195510)

[Supplementary Figure 9 – Funnel plot of studies reporting the prevalence of low sense of personal accomplishment among gastroenterologists and endoscopists 17](#_Toc191195511)

[Supplementary Figure 10 – Forest plot showing the odds ratio of burnout in female gastroenterologists and endoscopists in studies reporting gender-specific burnout rates 18](#_Toc191195512)

[Supplementary Figure 11 – Funnel plot of studies reporting gender-specific burnout rates and the odds ratio of burnout in female gastroenterologists and endoscopists 18](#_Toc191195513)

[Supplementary Table 1 – Summary of potential interventions for burnout and their outcomes 19](#_Toc191195514)

[References 27](#_Toc191195515)

#

# PRISMA Checklist

| **Section and Topic** | **Item #** | **Checklist item** | **Location where item is reported** |
| --- | --- | --- | --- |
| **TITLE** | | |  |
| Title | 1 | Identify the report as a systematic review. | Page 1 |
| **ABSTRACT** | | |  |
| Abstract | 2 | See the PRISMA 2020 for Abstracts checklist. | Page 3 - 4 |
| **INTRODUCTION** | | |  |
| Rationale | 3 | Describe the rationale for the review in the context of existing knowledge. | Page 6 |
| Objectives | 4 | Provide an explicit statement of the objective(s) or question(s) the review addresses. | Page 6 – 7 |
| **METHODS** | | |  |
| Eligibility criteria | 5 | Specify the inclusion and exclusion criteria for the review and how studies were grouped for the syntheses. | Page 8 – 9 |
| Information sources | 6 | Specify all databases, registers, websites, organisations, reference lists and other sources searched or consulted to identify studies. Specify the date when each source was last searched or consulted. | Page 8 |
| Search strategy | 7 | Present the full search strategies for all databases, registers and websites, including any filters and limits used. | Page S6 – S8 |
| Selection process | 8 | Specify the methods used to decide whether a study met the inclusion criteria of the review, including how many reviewers screened each record and each report retrieved, whether they worked independently, and if applicable, details of automation tools used in the process. | Page 8 – 9 |
| Data collection process | 9 | Specify the methods used to collect data from reports, including how many reviewers collected data from each report, whether they worked independently, any processes for obtaining or confirming data from study investigators, and if applicable, details of automation tools used in the process. | Page 9 |
| Data items | 10a | List and define all outcomes for which data were sought. Specify whether all results that were compatible with each outcome domain in each study were sought (e.g. for all measures, time points, analyses), and if not, the methods used to decide which results to collect. | Page 9 |
|  | 10b | List and define all other variables for which data were sought (e.g. participant and intervention characteristics, funding sources). Describe any assumptions made about any missing or unclear information. | Page 9 |
| Study risk of bias assessment | 11 | Specify the methods used to assess risk of bias in the included studies, including details of the tool(s) used, how many reviewers assessed each study and whether they worked independently, and if applicable, details of automation tools used in the process. | Page 9 |
| Effect measures | 12 | Specify for each outcome the effect measure(s) (e.g. risk ratio, mean difference) used in the synthesis or presentation of results. | Page 9 - 10 |
| Synthesis methods | 13a | Describe the processes used to decide which studies were eligible for each synthesis (e.g. tabulating the study intervention characteristics and comparing against the planned groups for each synthesis (item #5)). | Page 9 – 10 |
|  | 13b | Describe any methods required to prepare the data for presentation or synthesis, such as handling of missing summary statistics, or data conversions. | Page 9 – 10 |
|  | 13c | Describe any methods used to tabulate or visually display results of individual studies and syntheses. | Page 9 – 10 |
|  | 13d | Describe any methods used to synthesize results and provide a rationale for the choice(s). If meta-analysis was performed, describe the model(s), method(s) to identify the presence and extent of statistical heterogeneity, and software package(s) used. | Page 9 – 10 |
|  | 13e | Describe any methods used to explore possible causes of heterogeneity among study results (e.g. subgroup analysis, meta-regression). | Page 10 |
|  | 13f | Describe any sensitivity analyses conducted to assess robustness of the synthesized results. | Page 10 |
| Reporting bias assessment | 14 | Describe any methods used to assess risk of bias due to missing results in a synthesis (arising from reporting biases). | Page 9 |
| Certainty assessment | 15 | Describe any methods used to assess certainty (or confidence) in the body of evidence for an outcome. | N/A |
| **RESULTS** | | |  |
| Study selection | 16a | Describe the results of the search and selection process, from the number of records identified in the search to the number of studies included in the review, ideally using a flow diagram. | Page 11 |
|  | 16b | Cite studies that might appear to meet the inclusion criteria, but which were excluded, and explain why they were excluded. | N/A |
| Study characteristics | 17 | Cite each included study and present its characteristics. | Page 11 |
| Risk of bias in studies | 18 | Present assessments of risk of bias for each included study. | Page 13, 23 – 25 |
| Results of individual studies | 19 | For all outcomes, present, for each study: (a) summary statistics for each group (where appropriate) and (b) an effect estimate and its precision (e.g. confidence/credible interval), ideally using structured tables or plots. | Page 11 – 13 |
| Results of syntheses | 20a | For each synthesis, briefly summarise the characteristics and risk of bias among contributing studies. | Page 11 – 13 |
|  | 20b | Present results of all statistical syntheses conducted. If meta-analysis was done, present for each the summary estimate and its precision (e.g. confidence/credible interval) and measures of statistical heterogeneity. If comparing groups, describe the direction of the effect. | Page 11 – 13 |
|  | 20c | Present results of all investigations of possible causes of heterogeneity among study results. | Page 11 – 13 |
|  | 20d | Present results of all sensitivity analyses conducted to assess the robustness of the synthesized results. | Page 11 – 12 |
| Reporting biases | 21 | Present assessments of risk of bias due to missing results (arising from reporting biases) for each synthesis assessed. | Page 13 |
| Certainty of evidence | 22 | Present assessments of certainty (or confidence) in the body of evidence for each outcome assessed. | N/A |
| **DISCUSSION** | | |  |
| Discussion | 23a | Provide a general interpretation of the results in the context of other evidence. | Page 14 – 15 |
|  | 23b | Discuss any limitations of the evidence included in the review. | Page 16 |
|  | 23c | Discuss any limitations of the review processes used. | Page 16 |
|  | 23d | Discuss implications of the results for practice, policy, and future research. | Page 16 |
| **OTHER INFORMATION** | | |  |
| Registration and protocol | 24a | Provide registration information for the review, including register name and registration number, or state that the review was not registered. | Page 8 |
|  | 24b | Indicate where the review protocol can be accessed, or state that a protocol was not prepared. | Page 8 |
|  | 24c | Describe and explain any amendments to information provided at registration or in the protocol. | Page 8 |
| Support | 25 | Describe sources of financial or non-financial support for the review, and the role of the funders or sponsors in the review. | Page 2 |
| Competing interests | 26 | Declare any competing interests of review authors. | Page 2 |
| Availability of data, code and other materials | 27 | Report which of the following are publicly available and where they can be found: template data collection forms; data extracted from included studies; data used for all analyses; analytic code; any other materials used in the review. | Page 2 |

From: Page MJ, McKenzie JE, Bossuyt PM, Boutron I, Hoffmann TC, Mulrow CD, et al. The PRISMA 2020 statement: an updated guideline for reporting systematic reviews. BMJ 2021;372:n71. doi: 10.1136/bmj.n71

# Search Strategy

## MEDLINE

1 burnout.mp. 30562

2 burn-out.mp. 1190

3 (occupation* adj2 stress*).mp. 8043

4 exhaustion.mp. 32486

5 (work adj2 demand*).mp. 3269

6 work life balance.mp. 3651

7 Depersonalisation.mp. 575

8 Cynicism.mp. 1117

9 1 or 2 or 3 or 4 or 5 or 6 or 7 or 8 70370

10 gastroenterolog*.mp. 38734

11 hepatolog*.mp. 9675

12 endoscop*.mp. 310250

13 10 or 11 or 12 346012

14 9 and 13 195

## Embase

1 burnout.mp. 40897

2 burn-out.mp. 2063

3 (occupation* adj2 stress*).mp. 5727

4 exhaustion.mp. 47855

5 (work adj2 demand*).mp. 3889

6 work life balance.mp. 5259

7 Depersonalisation.mp. 786

8 Cynicism.mp. 1329

9 1 or 2 or 3 or 4 or 5 or 6 or 7 or 8 94718

10 gastroenterolog*.mp. 114661

11 hepatolog*.mp. 24622

12 endoscop*.mp. 574012

13 10 or 11 or 12 667000

14 9 and 13 548

15 limit 14 to "remove medline records" 300

## Scopus

TITLE-ABS-KEY ( burnout OR "burn-out" OR ( occupation* W/2 stress* ) OR exhaustion OR ( work W/2 demand* ) OR "work life balance" OR depersonalisation OR cynicism ) AND TITLE-ABS-KEY ( gastroenterolog* OR hepatolog* OR endoscop* )

## PsycINFO

1 burnout.mp. 22492

2 burn-out.mp. 743

3 (occupation* adj2 stress*).mp. 27852

4 exhaustion.mp. 10538

5 (work adj2 demand*).mp. 2848

6 work life balance.mp. 3589

7 Depersonalisation.mp. 448

8 Cynicism.mp. 2539

9 1 or 2 or 3 or 4 or 5 or 6 or 7 or 8 49561

10 gastroenterolog*.mp. 873

11 hepatolog*.mp. 130

12 endoscop*.mp. 1277

13 10 or 11 or 12 2207

14 9 and 13 13

# Modified Newcastle-Ottawa scale

**Selection**

1. **Representativeness of the cohort**
2. Truly representative (1)
3. Somewhat representative (1)
4. Selected group (0)
5. No description (0)
6. **Sample size**
7. Satisfactory; >100 (1)
8. Non-satisfactory; <100 (0)
9. **Response rate**
10. Good; >20% (1)
11. Low; <20% (0)

**Comparability**

1. **Potential confounders investigated by subgroup or multivariate analyses**
2. Study controls for the most important factor (1)
3. Study controls for any additional factors (2)
4. Does not control for any key factors (0)

**Outcome**

1. **Burnout screening tool**
2. Validated tool (2)
3. Non-validated tool (1)
4. No description (0)
5. **Appropriate statistical analysis**
6. Yes (1)
7. No (0)

# Supplementary Figures

## Supplementary Figure 1 – Funnel plot of studies reporting the prevalence of burnout among gastroenterologists and endoscopists


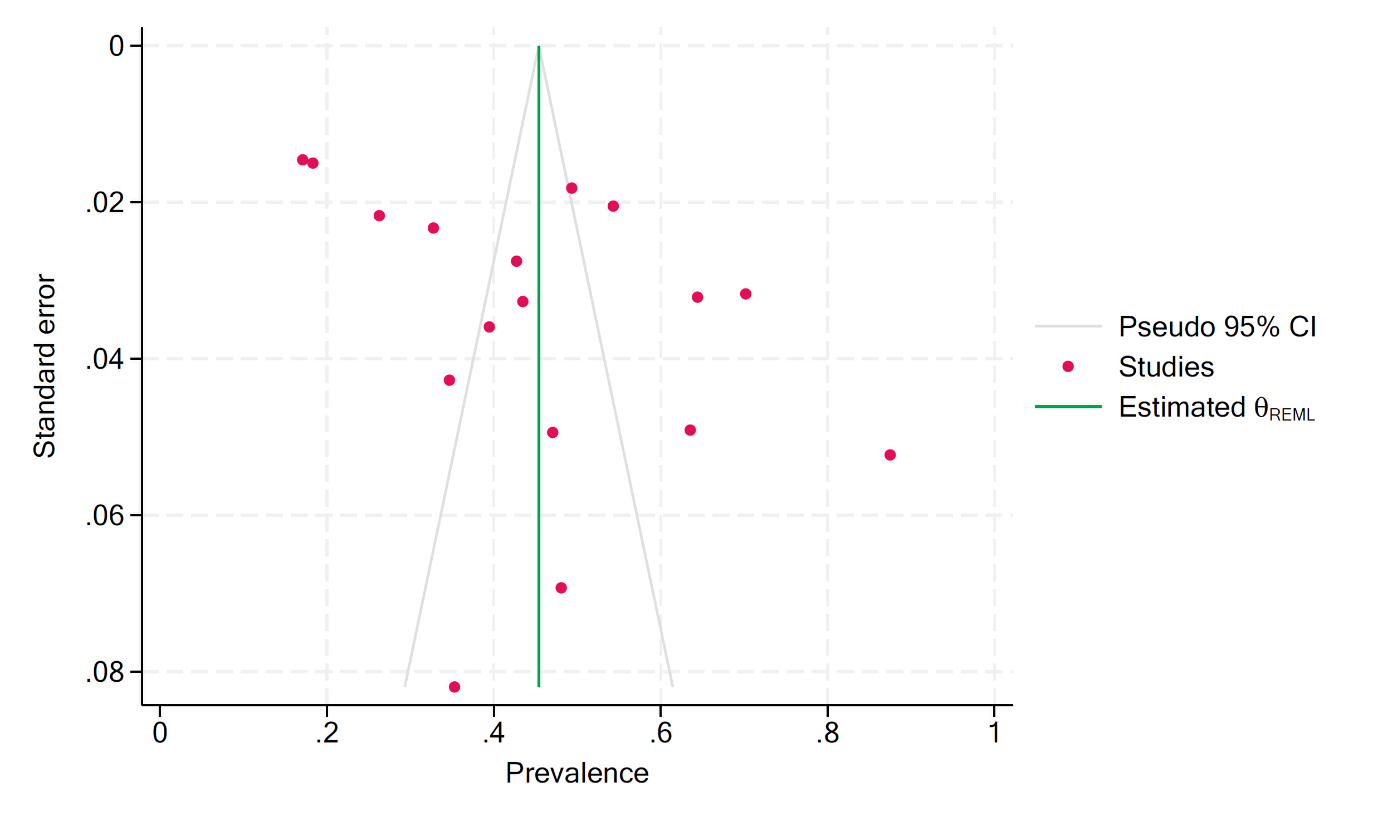


## Supplementary Figure 2 – Forest plot of studies reporting the prevalence of burnout sub-grouped according to the tool used for assessment


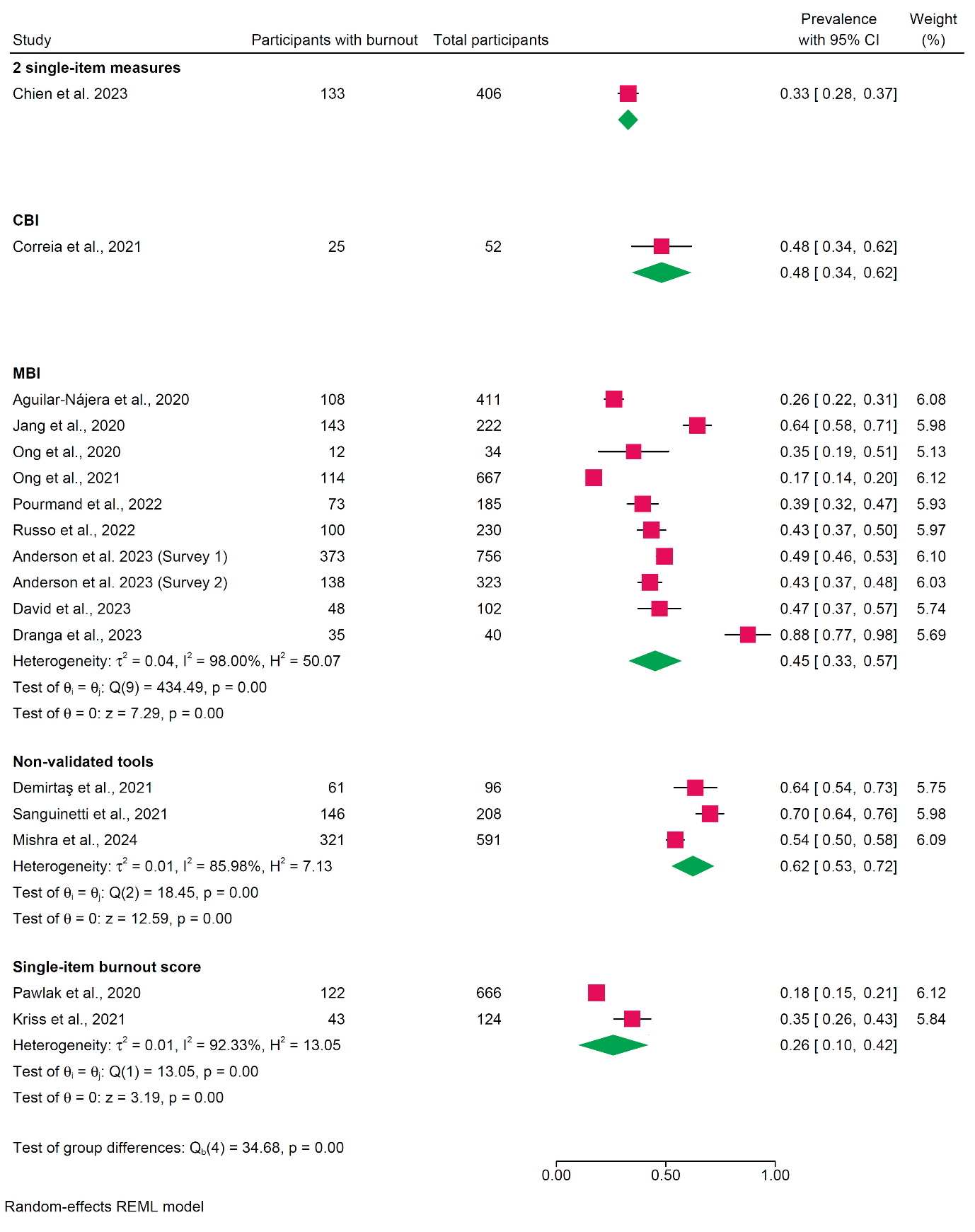


## Supplementary Figure 3 – Forest plot of studies reporting the prevalence of burnout excluding those using non-validated questionnaires


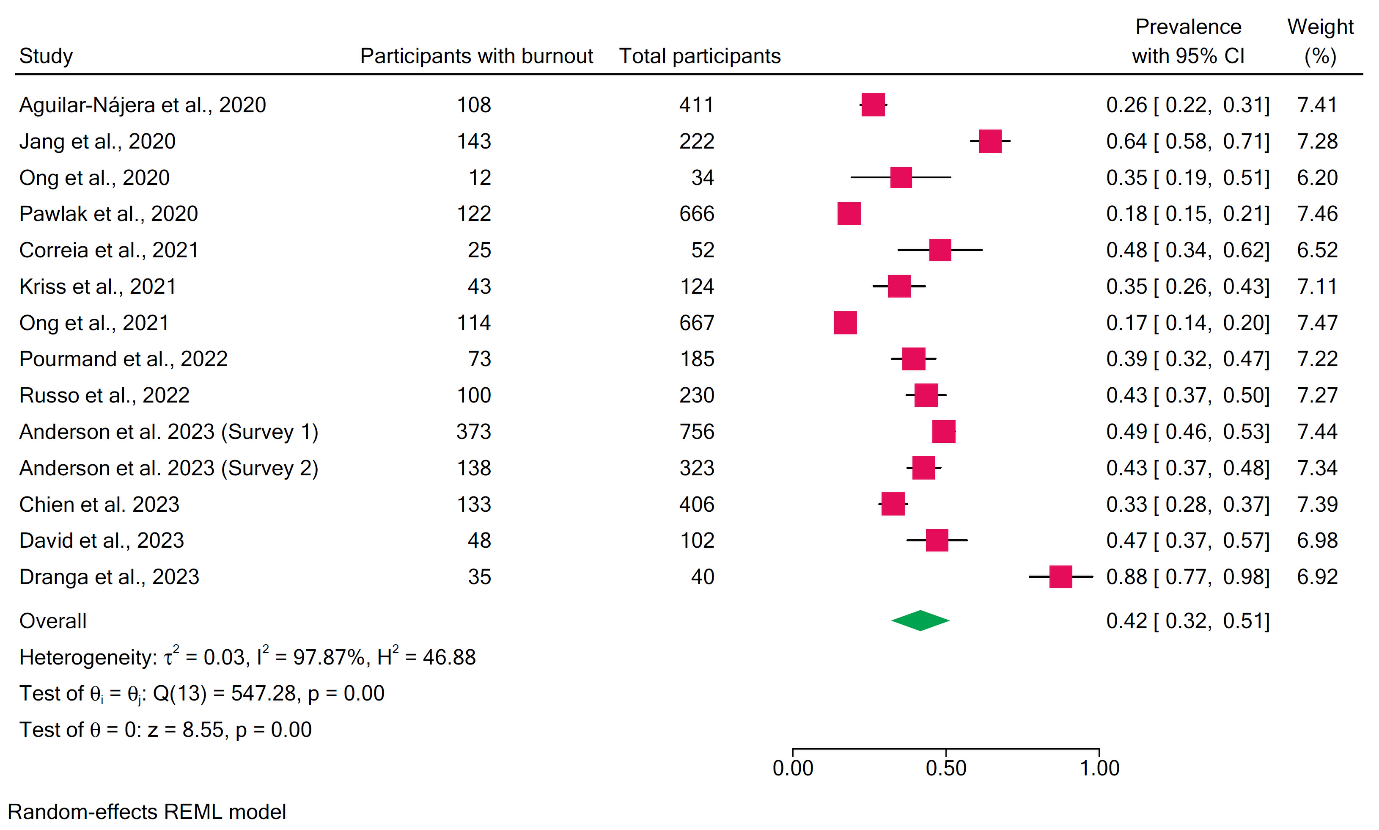


## Supplementary Figure 4 – Forest plot of studies reporting the prevalence of burnout sub-grouped according to response rates


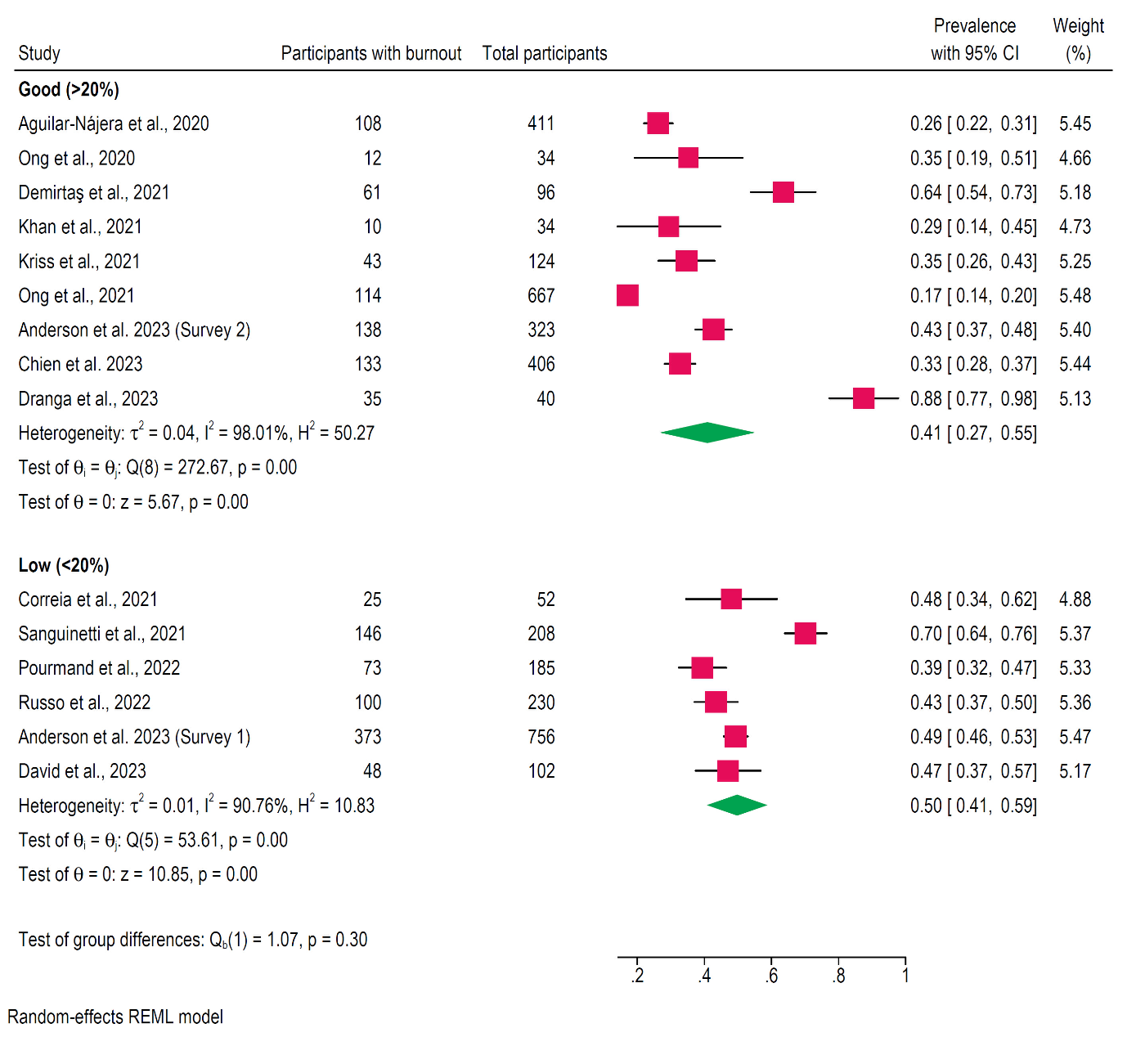


## Supplementary Figure 5 – Forest plot of studies reporting the prevalence of burnout sub-grouped according to participants’ career stage


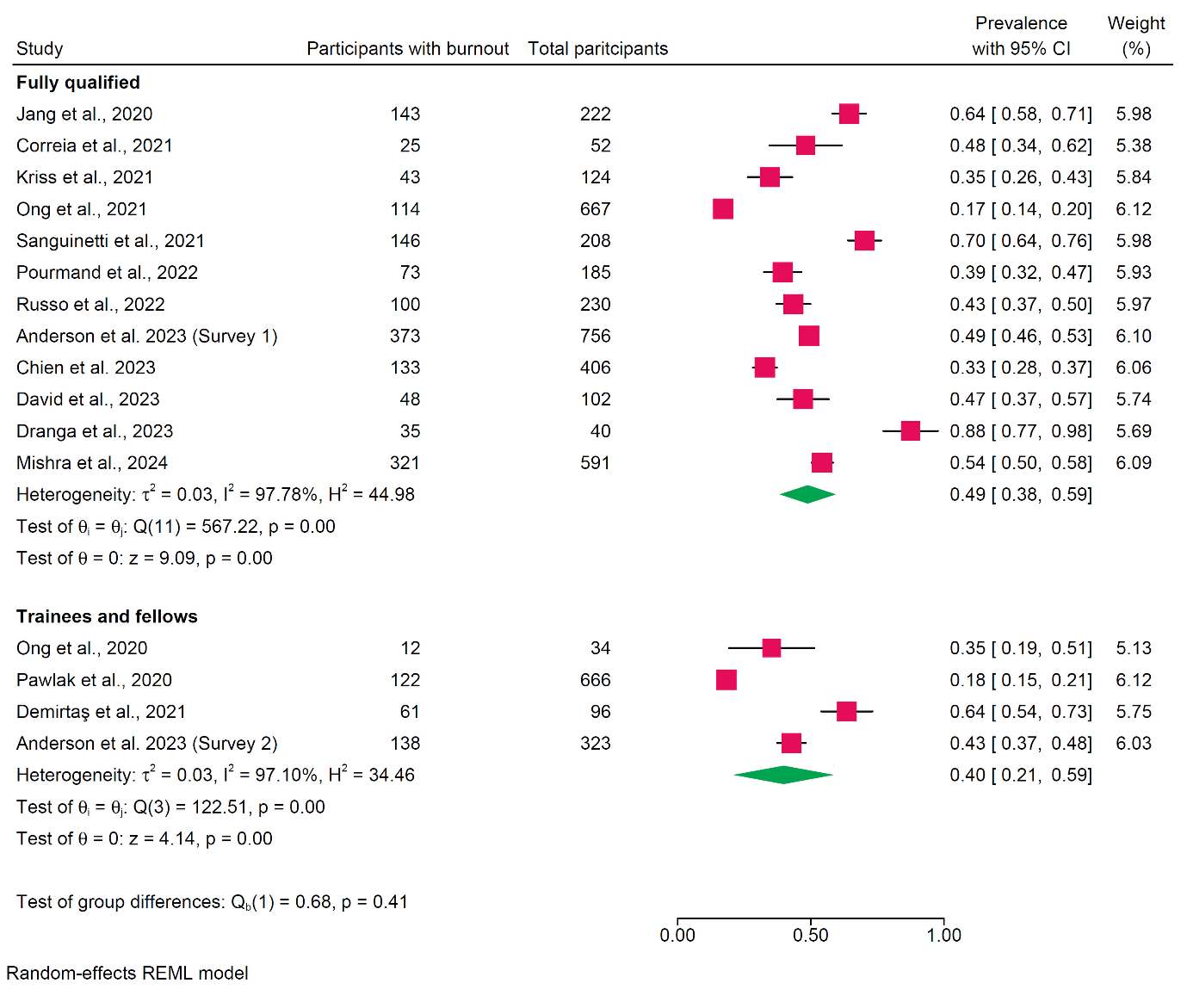


## Supplementary Figure 6 – Forest plot of studies reporting the prevalence of burnout sub-grouped according to the time of the survey


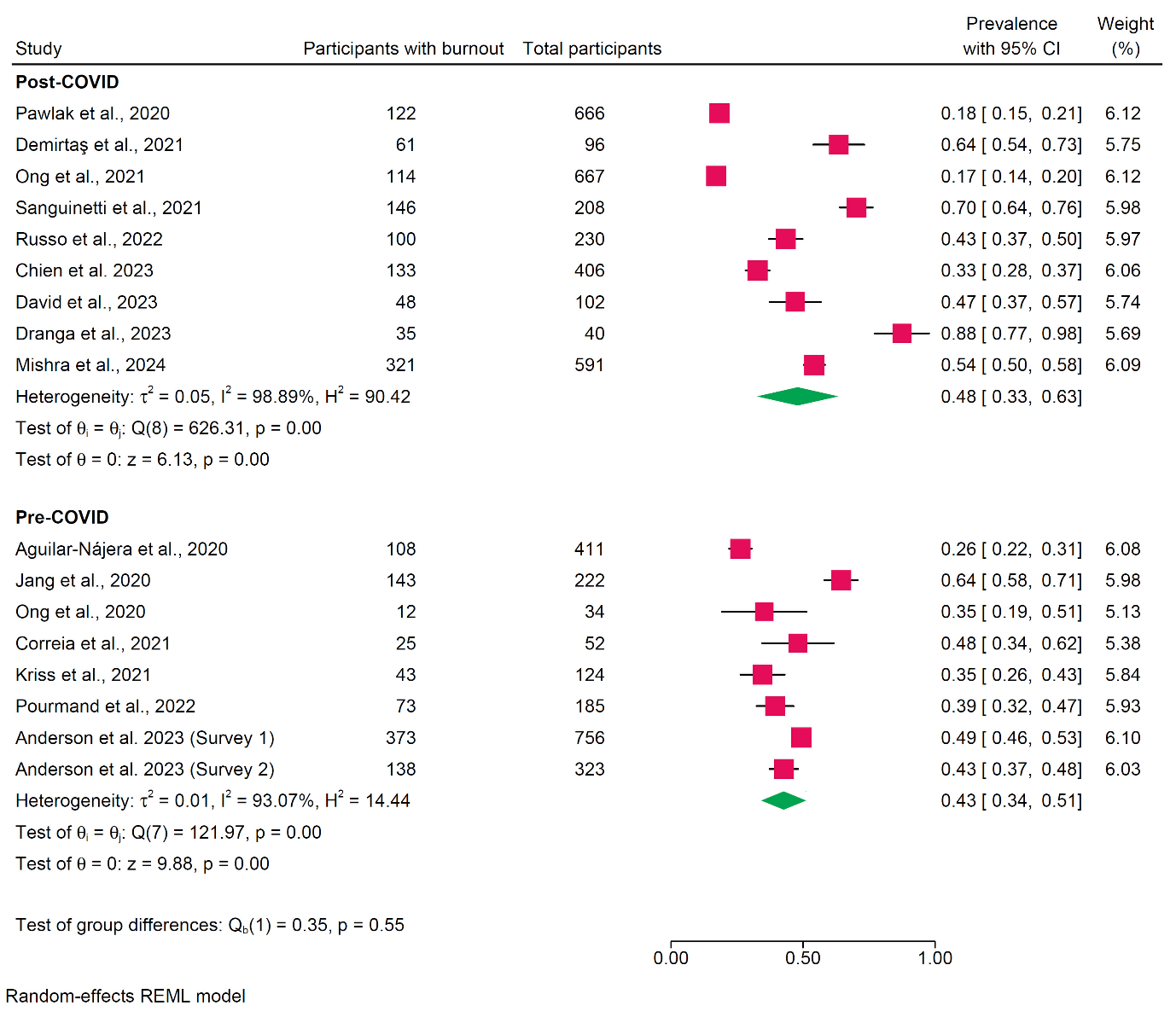


## Supplementary Figure 7 – Leave one out sensitivity analysis showing the pooled prevalence of burnout after excluding one study at a time


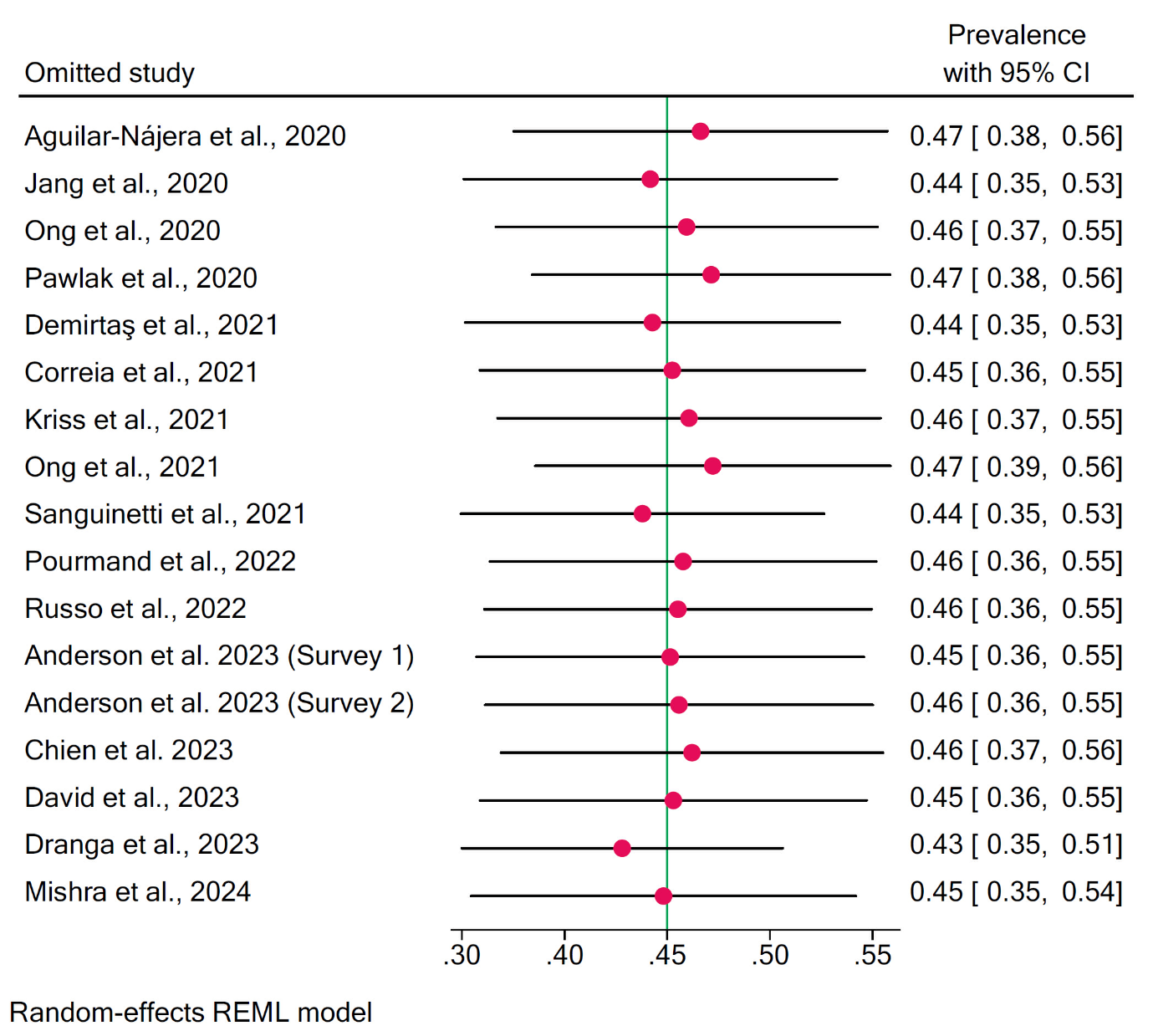


## Supplementary Figure 8 – Funnel plot of studies reporting the prevalence of emotional exhaustion among gastroenterologists and endoscopists


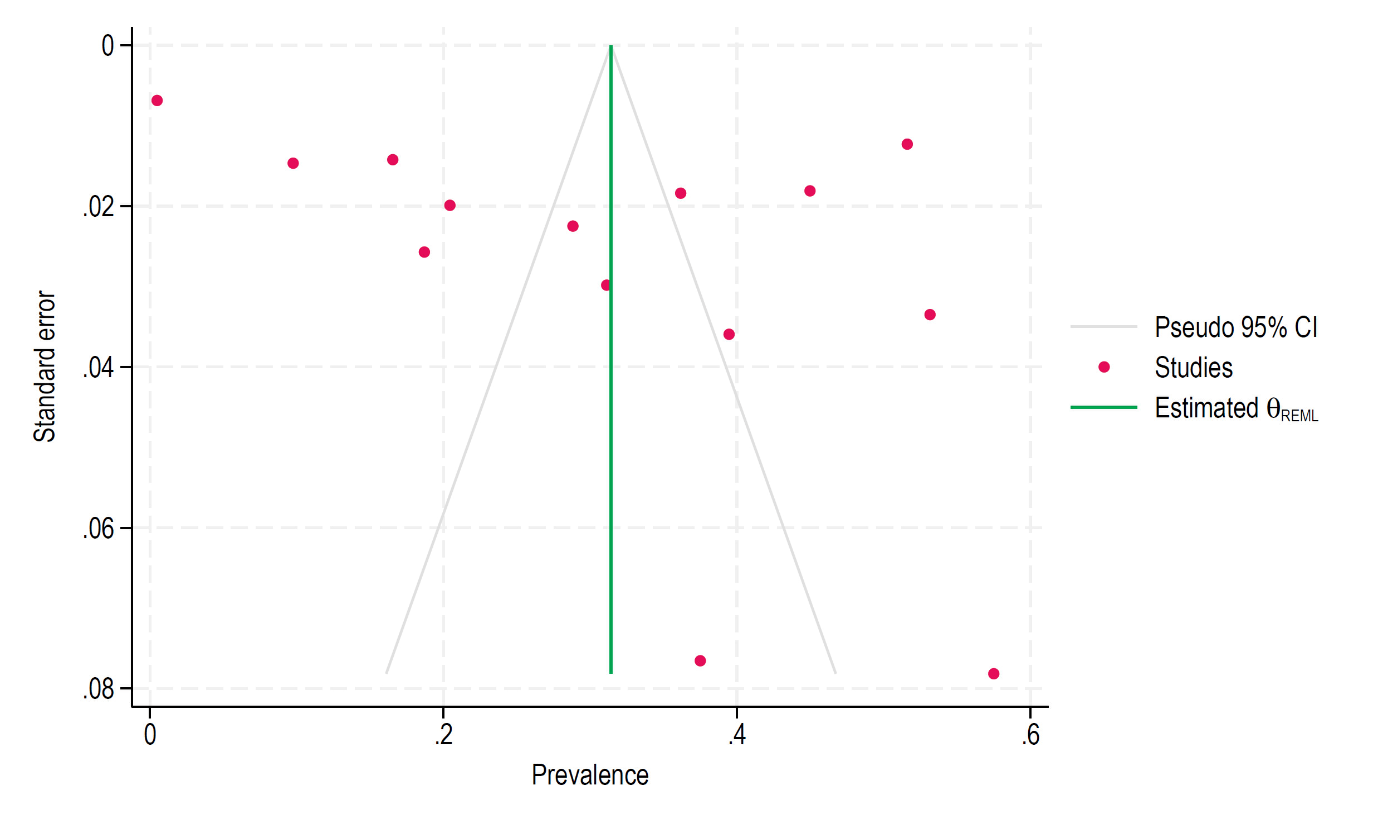


## Supplementary Figure 9 – Funnel plot of studies reporting the prevalence of low sense of personal accomplishment among gastroenterologists and endoscopists


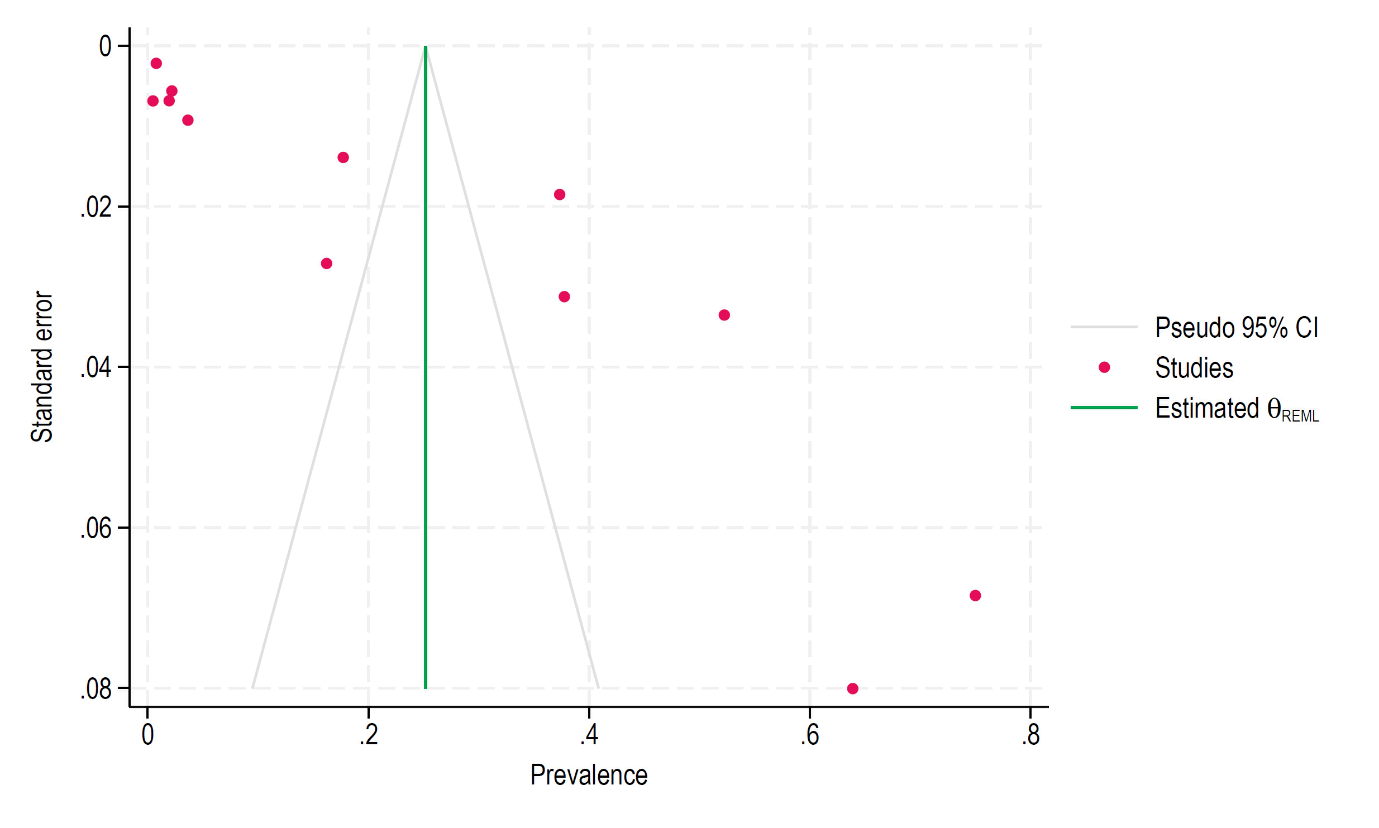


## Supplementary Figure 10 – Forest plot showing the odds ratio of burnout in female gastroenterologists and endoscopists in studies reporting gender-specific burnout rates


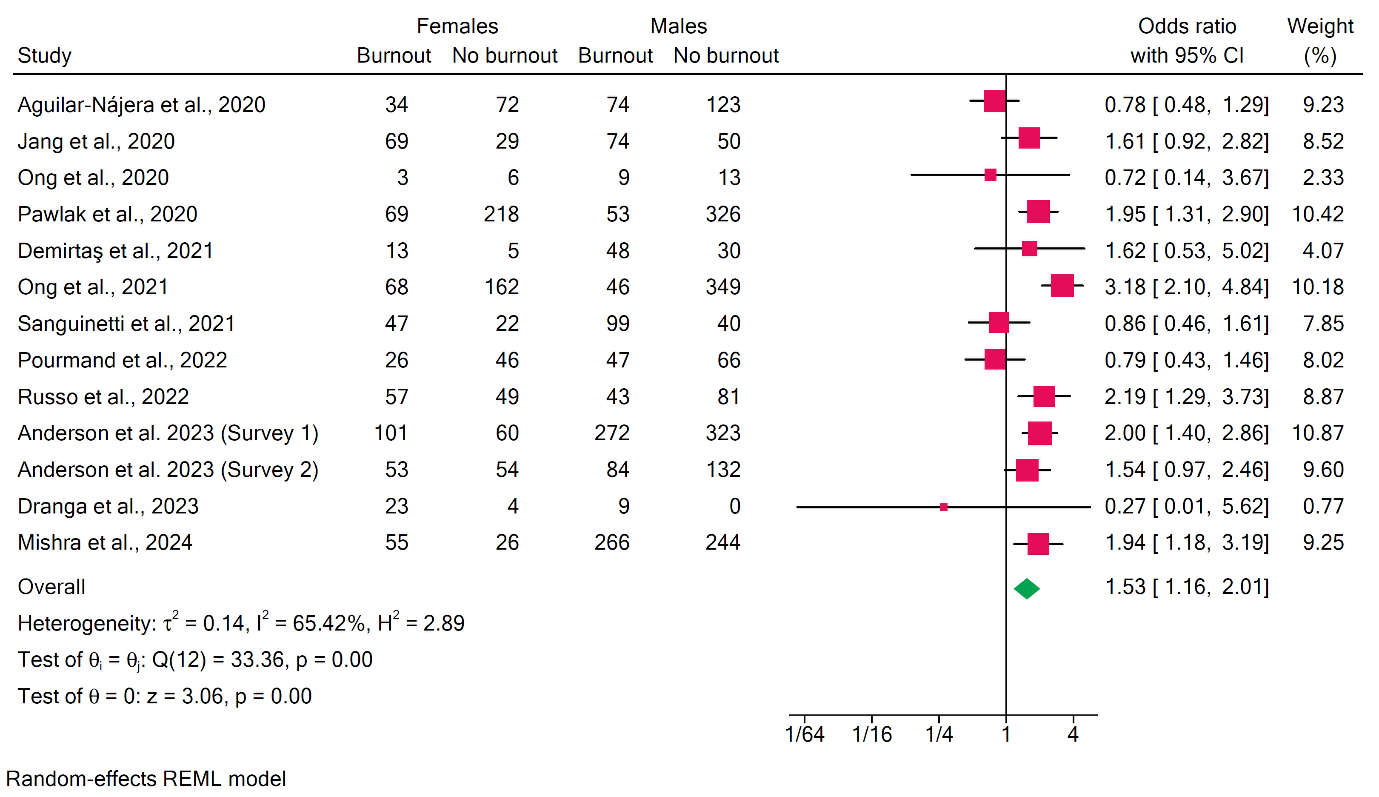


## Supplementary Figure 11 – Funnel plot of studies reporting gender-specific burnout rates and the odds ratio of burnout in female gastroenterologists and endoscopists


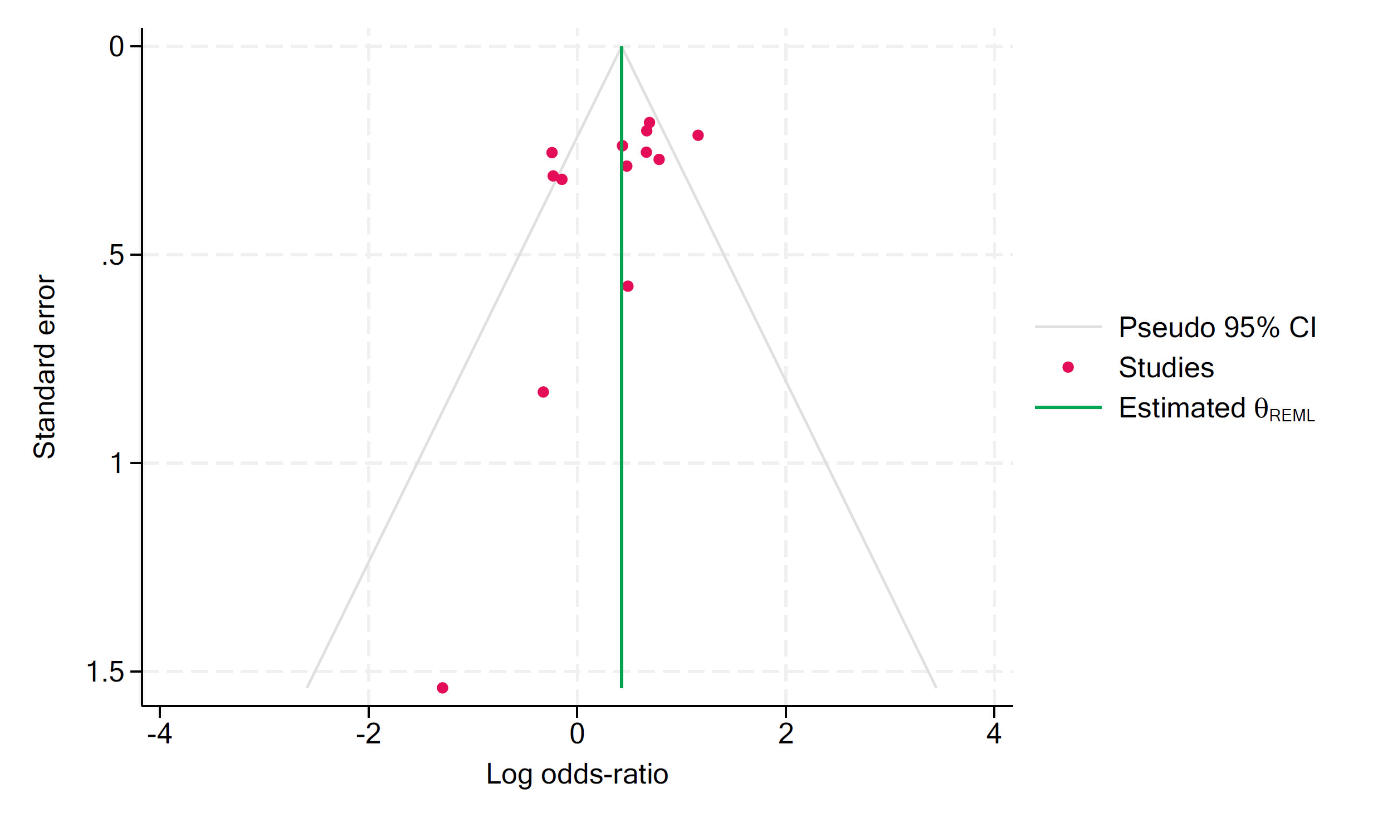


# Supplementary Table 1 – Summary of potential interventions for burnout and their outcomes

| Type of Intervention | Description of the intervention | Level of intervention | Outcomes | Study (Ref) |
| --- | --- | --- | --- | --- |
| Educational | Coaching sessions facilitated by a professional coach focusing on assessing needs, identifying values, setting goals, and creating an action plan. | Organisational/Individual | Reductions in emotional exhaustion scores and improvements in overall quality of life | Dyrbye et al., 2022 (1) |
|  | Web-based Implementation program to improve dimensions of wellbeing | Individual | Improved well-being, depressive symptoms, work-life integration, happiness, emotional thriving recovery. | Sexton et al., 2022 (2) |
|  | The efficacy of a program created a common language, culture, and leadership strategy to promote well-being. | Organisational | Emotional and work-related exhaustion decreased significantly | Dunn et al., 2007 (3) |
|  | Self-administered psycho-  therapeutic tool | Individual | No significant differences between  the groups | Milstein et al.,  2009 (4) |
|  | Team-based burnout intervention program combining a staff support group with a participatory research approach | Individual | Significant reduction of exhaustion  and depersonalisation | Le Blanc et  al., 2007  (5) |
|  | Counselling (lasting one day (individual) or one week (group based)) aimed at motivating reflection on and acknowledgement of the doctors’ situation and personal needs | Individual | A significant reduction in the exhaustion dimension was associated with reduced working hours | Rø KE et al.,  2008 (6) |
|  | Intensive educational program in mindfulness, communication, and self-awareness to improve primary care physicians' well-being, psychological distress, burnout, and capacity for relating to patients | Individual | Significant reduction in burnout | Krasner MS, 2009 (7) |
|  | Communication and stress management skills training programme on medical residents' self-efficacy to communicate and to manage stress in interviews, stress to communicate in interviews, and burnout | Individual | No significant changes were noted in burnout | Bragard et al.,  2010 (8) |
|  | Stress management workshop designed for physicians | Individual | Significant reduction in exhaustion  dimension | McCue et al.,  1991  (9) |
|  | Communication skills training (CST) program targeting burnout and evaluating its impact on physician behaviour. | Individual | No reductions in burnout levels | Butow P. et al.  2008 (10) |
|  | Two self-care workshops coordinated by mental health professionals, who addressed  aspects of burnout syndrome, such as the identification of risk  factors, coping behaviours, preventive behaviours, and  self-care | Individual | A brief intervention was not effective in reducing burnout prevalence despite the achievement of an improvement in depersonalisation | Martins  et al., 2011 (11) |
|  | The intervention taught physicians how to deal with stress and burnout, patients’ death, and distress. | Individual | The intervention significantly decreased doctors’ levels of burnout (e.g. emotional exhaustion and depersonalisation) and anxiety. | Medisaukaite and Kamau, 2019  (12) |
|  | A 6-month, web-based group-coaching program, Better Together Physician Coaching, developed and facilitated by trained life coaches and physicians. | Individual | Professional coaching reduced emotional exhaustion and impostor syndrome scores and increased self-compassion scores among female resident physicians. | Fainstad et al., 2022  (13) |
| Mindfulness/meditation | Mindfulness exercises and interventions (purposefully paying attention moment by moment, in a non-judgmental way) | Individual | Fewer burnout symptoms and increased work engagement and wellbeing | Verweij et al., 2016 (14) |
|  | Respiratory One Method  (a form of meditation that relies on verbalising the  the word "one" during exhalation and is designed to mitigate the  impact of emotional arousal) as a burnout risk reduction method | Individual | Significant reduction in exhaustion  dimension | Ospina-Kammerer et al., 2003 (15) |
|  | Mindfulness-based stress  programme, weekly  mindfulness exercise, a weekly group reflection  about the weekly topic and the mindfulness exercise | Individual | The intervention helped reduce and control risks of developing burnout and cardiovascular disease in this population and enhanced well-being | Amutio et al., 2015 (16) |
|  | Art therapy and cognitive behavioural therapy | Individual | Significant reduction of burnout | Italia S et al.,  2008  (17) |
|  | Facilitated  discussion groups incorporating elements of mindfulness,  reflection, shared experience, and small-group learning for  9 mo. The institution provided protected time for participants. | Organisational | The intervention improved meaning and engagement in work and reduced depersonalisation, with sustained results at 12 months after the study. | West et al.,  2014  (18) |
|  | Effectiveness of a phone-based meditation application in reducing stress, depression, anxiety and burnout among nurses and physicians after 3 months of weekly use. | Individual | The usefulness of phone-based mindfulness meditation helped reduce stress, depression, anxiety and burnout among emergency medicine personnel. | Lambert et al., 2020  (19) |
|  | Effectiveness of yoga-based mind-body interventions called RISE (resilience, integration, self-awareness, engagement) for residents of multiple specialities and academic medical centres consisting of 6 weekly sessions with suggested home practice | individual | The intervention improved mindfulness, stress, burnout, and physician well-being from baseline to post-program, which were sustained at two-month follow-up. | Loewenthal et al., 2021  (20) |
|  | Enhanced stress resilience training, an iteratively adapted and tailored mindfulness-based cognitive training program, was administered | Individual | ESRT can benefit executive function, burnout, and physiological distress in post-graduate year 1 residents, with the potential for tailoring to optimise effects. | Lebares et al., 2021  (21) |
| Schedule change | Discussion between clinical and research staff about a list of topics/interventions to address adverse clinician work conditions | Organisational | Organisations may improve burnout, dissatisfaction and retention by addressing communication and targeting clinician concerns. | Linzer et al., 2015 (22) |
|  | The relationship between burnout and professional satisfaction with changes in physicians' professional efforts was evaluated | Organisational | Burnout and decline in satisfaction  were strongly associated with actual  reductions in professional work  effort over the following 24 months | Shanafelt et al.,  2016  (23) |
|  | Two intensivists staffing daily coverage by a single intensivist in half-month rotations were compared with weekday coverage by a single intensivist, with weekend cross-coverage by colleagues. | Organisational | Work schedules where intensivists received weekend breaks were better for the physicians and, despite lower continuity of intensivist care, did not worsen outcomes for medical ICU patients. | Ali et al., 2011 (24) |
|  | Residents in 2 university-affiliated ICUs were randomly  assigned to in-house overnight schedules of 12 | Organisational | The findings do not support the purported advantages of shorter duty schedules | Parshuram  et al., 2015  (25) |
|  | Trial of two intensivist staffing models, performed in ICUs, alternated 8-week blocks of two staffing models: the standard model, where one intensivist worked for 7 days, taking night calls from home, and the shift work model, where one intensivist worked 7-day shifts, while other intensivists remained in the ICU at night. | Organisational | Shiftwork staffing was better for intensivists, and most were receptive once they had experienced it | Garland et al., 2012  (26) |
|  | A 5-h period of protected time in which interns were  expected to sleep | Organisational | A protected sleep period produced few consistent improvements in depression, burnout, or empathy, although depression was already low at baseline. | Shea et al.,  2014  (27) |
|  | Assignment to random sequences of 2-week shift rotations on unplanned patient revisits | Organisational | The intervention was associated with better self-rated measures of attending physician burnout and emotional exhaustion but worse evaluations by trainees. | Lucas et al., 2012  (28) |
| Discussion Group | Three Debriefing sessions and a focus group that explored themes around work-related stressors,  coping mechanisms, and potential strategies to improve  junior medical officers' well-being | Individual | The intervention was well-received but there was no significant difference in burnout scores with debriefing | Gunasingam  et al., 2015 (29) |
|  | Bimonthly groups who met  regularly with trained discussion group leaders to discuss  topics related to stress, balance, and job satisfaction | Individual | Facilitated group discussion intervention did not decrease burnout in resident physicians | Ripp et al., 2016 (30) |
|  | Twelve biweekly self-facilitated discussion groups involving reflection, shared experience, and small-group learning took place over 6 months. | Individual | Self-facilitated physician small-group meetings improved burnout, depressive symptoms, and job satisfaction. | West et al., 2021  (31) |
| Physical exercise | Physical activities to improve physical fitness and reduce stress | Individual | Lower burnout rates, higher PA and improvement in QoL | Weight et al., 2013 (32) |
| Pharmacological intervention | Cannabidiol (CBD) plus standard care compared with those receiving standard care alone. | individual | CBD therapy reduced burnout and emotional exhaustion symptoms among healthcare professionals working with patients during the COVID-19 pandemic. | Crippa et al., 2021  (33) |

## References

1. Dyrbye LN, Shanafelt TD, Gill PR, Satele D V., West CP. Effect of a Professional Coaching Intervention on the Well-being and Distress of Physicians. JAMA Intern Med [Internet]. 2019 Oct 1;179(10):1406. Available from: https://jamanetwork.com/journals/jamainternalmedicine/fullarticle/2740206

2. Sexton JB, Adair KC, Cui X, Tawfik DS, Profit J. Effectiveness of a bite-sized web-based intervention to improve healthcare worker wellbeing: A randomized clinical trial of WISER. Front Public Heal [Internet]. 2022 Dec 8;10. Available from: https://www.frontiersin.org/articles/10.3389/fpubh.2022.1016407/full

3. Dunn PM, Arnetz BB, Christensen JF, Homer L. Meeting the Imperative to Improve Physician Well-being: Assessment of an Innovative Program. J Gen Intern Med [Internet]. 2007 Oct 12;22(11):1544–52. Available from: http://link.springer.com/10.1007/s11606-007-0363-5

4. Milstein JM, Raingruber BJ, Bennett SH, Kon AA, Winn CA, Paterniti DA. Burnout assessment in house officers: Evaluation of an intervention to reduce stress. Med Teach [Internet]. 2009 Jan 3;31(4):338–41. Available from: http://www.tandfonline.com/doi/full/10.1080/01421590802208552

5. Le Blanc PM, Hox JJ, Schaufeli WB, Taris TW, Peeters MCW. Take care! The evaluation of a team-based burnout intervention program for oncology care providers. J Appl Psychol [Internet]. 2007;92(1):213–27. Available from: https://doi.apa.org/doi/10.1037/0021-9010.92.1.213

6. Ro KEI, Gude T, Tyssen R, Aasland OG. Counselling for burnout in Norwegian doctors: one year cohort study. BMJ [Internet]. 2008 Nov 11;337(nov11 3):a2004–a2004. Available from: https://www.bmj.com/lookup/doi/10.1136/bmj.a2004

7. Krasner MS. Association of an Educational Program in Mindful Communication With Burnout, Empathy, and Attitudes Among Primary Care Physicians. JAMA [Internet]. 2009 Sep 23;302(12):1284. Available from: http://jama.jamanetwork.com/article.aspx?doi=10.1001/jama.2009.1384

8. Bragard I, Etienne A-M, Merckaert I, Libert Y, Razavi D. Efficacy of a Communication and Stress Management Training on Medical Residents’ Self-efficacy, Stress to Communicate and Burnout. J Health Psychol [Internet]. 2010 Oct 7;15(7):1075–81. Available from: https://journals.sagepub.com/doi/10.1177/1359105310361992

9. McCue JD, Sachs CL. A stress management workshop improves residents’ coping skills. Arch Intern Med [Internet]. 1991 Nov;151(11):2273–7. Available from: http://www.ncbi.nlm.nih.gov/pubmed/1953233

10. Butow P, Cockburn J, Girgis A, Bowman D, Schofield P, D’Este C, et al. Increasing oncologists’ skills in eliciting and responding to emotional cues: evaluation of a communication skills training program. Psychooncology [Internet]. 2008 Mar 18;17(3):209–18. Available from: https://onlinelibrary.wiley.com/doi/10.1002/pon.1217

11. Martins AE, Davenport MC, Valle M de la P Del, Lalla S Di, Domínguez P, Ormando L, et al. Impact of a brief intervention on the burnout levels of pediatric residents. J Pediatr (Rio J) [Internet]. 2011 Dec 12;87(6):493–8. Available from: http://www.jped.com.br/Redirect.aspx?varArtigo=2266

12. Medisauskaite A, Kamau C. Reducing burnout and anxiety among doctors: Randomized controlled trial. Psychiatry Res [Internet]. 2019 Apr;274:383–90. Available from: https://linkinghub.elsevier.com/retrieve/pii/S0165178119301751

13. Fainstad T, Mann A, Suresh K, Shah P, Dieujuste N, Thurmon K, et al. Effect of a Novel Online Group-Coaching Program to Reduce Burnout in Female Resident Physicians. JAMA Netw Open [Internet]. 2022 May 6;5(5):e2210752. Available from: https://jamanetwork.com/journals/jamanetworkopen/fullarticle/2791968

14. Verweij H, Waumans RC, Smeijers D, Lucassen PL, Donders ART, van der Horst HE, et al. Mindfulness-based stress reduction for GPs: results of a controlled mixed methods pilot study in Dutch primary care. Br J Gen Pract [Internet]. 2016 Feb;66(643):e99–105. Available from: https://bjgp.org/lookup/doi/10.3399/bjgp16X683497

15. Ospina-Kammerer V, Figley CR. An evaluation of the Respiratory One Method (ROM) in reducing emotional exhaustion among family physician residents. Int J Emerg Ment Health [Internet]. 2003;5(1):29–32. Available from: http://www.ncbi.nlm.nih.gov/pubmed/12722487

16. Amutio A, Martínez-Taboada C, Delgado LC, Hermosilla D, Mozaz MJ. Acceptability and Effectiveness of a Long-Term Educational Intervention to Reduce Physicians’ Stress-Related Conditions. J Contin Educ Health Prof [Internet]. 2015;35(4):255–60. Available from: https://journals.lww.com/00005141-201535040-00004

17. Italia S, Favara‐Scacco C, Di Cataldo A, Russo G. Evaluation and art therapy treatment of the burnout syndrome in oncology units. Psychooncology [Internet]. 2008 Jul 9;17(7):676–80. Available from: https://onlinelibrary.wiley.com/doi/10.1002/pon.1293

18. West CP, Dyrbye LN, Rabatin JT, Call TG, Davidson JH, Multari A, et al. Intervention to Promote Physician Well-being, Job Satisfaction, and Professionalism. JAMA Intern Med [Internet]. 2014 Apr 1;174(4):527. Available from: http://archinte.jamanetwork.com/article.aspx?doi=10.1001/jamainternmed.2013.14387

19. Lambert KG, Aufricht WR, Mudie D, Brown LH. Does a phone-based meditation application improve mental wellness in emergency medicine personnel? Am J Emerg Med [Internet]. 2020 Dec;38(12):2740–1. Available from: https://linkinghub.elsevier.com/retrieve/pii/S0735675720302886

20. Loewenthal J, Dyer NL, Lipsyc-Sharf M, Borden S, Mehta DH, Dusek JA, et al. Evaluation of a Yoga-Based Mind-Body Intervention for Resident Physicians: A Randomized Clinical Trial. Glob Adv Heal Med [Internet]. 2021 Jan 10;10. Available from: https://journals.sagepub.com/doi/10.1177/21649561211001038

21. Lebares CC, Coaston TN, Delucchi KL, Guvva E V., Shen WT, Staffaroni AM, et al. Enhanced Stress Resilience Training in Surgeons. Ann Surg [Internet]. 2021 Mar;273(3):424–32. Available from: https://journals.lww.com/10.1097/SLA.0000000000004145

22. Linzer M, Poplau S, Grossman E, Varkey A, Yale S, Williams E, et al. A Cluster Randomized Trial of Interventions to Improve Work Conditions and Clinician Burnout in Primary Care: Results from the Healthy Work Place (HWP) Study. J Gen Intern Med [Internet]. 2015 Aug 28;30(8):1105–11. Available from: http://link.springer.com/10.1007/s11606-015-3235-4

23. Shanafelt TD, Mungo M, Schmitgen J, Storz KA, Reeves D, Hayes SN, et al. Longitudinal Study Evaluating the Association Between Physician Burnout and Changes in Professional Work Effort. Mayo Clin Proc [Internet]. 2016 Apr;91(4):422–31. Available from: https://linkinghub.elsevier.com/retrieve/pii/S0025619616001014

24. Ali NA, Wolf KM, Hammersley J, Hoffmann SP, O’Brien JM, Phillips GS, et al. Continuity of Care in Intensive Care Units. Am J Respir Crit Care Med [Internet]. 2011 Oct 1;184(7):803–8. Available from: https://www.atsjournals.org/doi/10.1164/rccm.201103-0555OC

25. Parshuram CS, Amaral ACKB, Ferguson ND, Baker GR, Etchells EE, Flintoft V, et al. Patient safety, resident well-being and continuity of care with different resident duty schedules in the intensive care unit: a randomized trial. Can Med Assoc J [Internet]. 2015 Mar 17;187(5):321–9. Available from: http://www.cmaj.ca/lookup/doi/10.1503/cmaj.140752

26. Garland A, Roberts D, Graff L. Twenty-four–Hour Intensivist Presence. Am J Respir Crit Care Med [Internet]. 2012 Apr 1;185(7):738–43. Available from: https://www.atsjournals.org/doi/10.1164/rccm.201109-1734OC

27. Shea JA, Bellini LM, Dinges DF, Curtis ML, Tao Y, Zhu J, et al. Impact of Protected Sleep Period for Internal Medicine Interns on Overnight Call on Depression, Burnout, and Empathy. J Grad Med Educ [Internet]. 2014 Jun 1;6(2):256–63. Available from: https://meridian.allenpress.com/jgme/article/6/2/256/34503/Impact-of-Protected-Sleep-Period-for-Internal

28. Lucas BP, Trick WE, Evans AT, Mba B, Smith J, Das K, et al. Effects of 2- vs 4-Week Attending Physician Inpatient Rotations on Unplanned Patient Revisits, Evaluations by Trainees, and Attending Physician Burnout. JAMA [Internet]. 2012 Dec 5;308(21):2199. Available from: http://jama.jamanetwork.com/article.aspx?doi=10.1001/jama.2012.36522

29. Gunasingam N, Burns K, Edwards J, Dinh M, Walton M. Reducing stress and burnout in junior doctors: the impact of debriefing sessions. Postgrad Med J [Internet]. 2015 Apr 1;91(1074):182–7. Available from: https://academic.oup.com/pmj/article/91/1074/182/6993864

30. Ripp JA, Fallar R, Korenstein D. A Randomized Controlled Trial to Decrease Job Burnout in First-Year Internal Medicine Residents Using a Facilitated Discussion Group Intervention. J Grad Med Educ [Internet]. 2016 May 1;8(2):256–9. Available from: https://meridian.allenpress.com/jgme/article/8/2/256/34479/A-Randomized-Controlled-Trial-to-Decrease-Job

31. West CP, Dyrbye LN, Satele D V., Shanafelt TD. Colleagues Meeting to Promote and Sustain Satisfaction (COMPASS) Groups for Physician Well-Being. Mayo Clin Proc [Internet]. 2021 Oct;96(10):2606–14. Available from: https://linkinghub.elsevier.com/retrieve/pii/S0025619621002573

32. Weight CJ, Sellon JL, Lessard-Anderson CR, Shanafelt TD, Olsen KD, Laskowski ER. Physical Activity, Quality of Life, and Burnout Among Physician Trainees: The Effect of a Team-Based, Incentivized Exercise Program. Mayo Clin Proc [Internet]. 2013 Dec;88(12):1435–42. Available from: https://linkinghub.elsevier.com/retrieve/pii/S002561961300829X

33. Crippa JAS, Zuardi AW, Guimarães FS, Campos AC, de Lima Osório F, Loureiro SR, et al. Efficacy and Safety of Cannabidiol Plus Standard Care vs Standard Care Alone for the Treatment of Emotional Exhaustion and Burnout Among Frontline Health Care Workers During the COVID-19 Pandemic. JAMA Netw Open [Internet]. 2021 Aug 13;4(8):e2120603. Available from: https://jamanetwork.com/journals/jamanetworkopen/fullarticle/2782994
